# Patient and Public Involvement and Engagement (PPIE): first steps in the process of the engagement in research projects in Brazil

**DOI:** 10.1590/1414-431X2022e12369

**Published:** 2022-07-25

**Authors:** I.M. Bensenor, A.C. Goulart, G.N. Thomas, G.Y.H. Lip, on behalf of the NIHR Global Health Research Group on Atrial Fibrillation Management

**Affiliations:** 1 Departamento de Clínica Médica, Faculdade de Medicina, Universidade de São Paulo, São Paulo, SP, Brasil; 2 Centro de Pesquisa Clínica e Epidemiológica, Hospital Universitário, Universidade de São Paulo, São Paulo, SP, Brasil; 3 Institute of Applied Health Research, University of Birmingham, Birmingham, United Kingdom; 4 Liverpool Centre for Cardiovascular Science, University of Liverpool, Liverpool, United Kingdom

**Keywords:** Patient and Public Involvement and Engagement, First process, Engagements, Research projects

## Abstract

Patient and Public Involvement and Engagement (PPIE) – sometimes called Community Engagement and Involvement (CEI) – comes as a big challenge but one that can be very helpful for health care professionals and stakeholders in planning better health policies for attending to the main needs of the community. PPIE involves three pillars: public involvement, public engagement, and participation. Public involvement occurs when members of the general population are actively involved in developing the research question, designing, and conducting the research. Public engagement tells people about new studies, why they are important, the impact of results, the possible implication of the main findings for the community, and the possible impact of these new findings in society, as well as, in the dissemination of knowledge to the general population. Participation is being a volunteer in the study. Our experience with PPIE, to the best of our knowledge the first initiative in Brazil, is a partnership with the University of Birmingham, the University of Liverpool, and the NIHR Global Health Group on Atrial Fibrillation (AF) Management focusing on the AF care pathway exploring the important aspects of diagnosis and treatment in the primary care system from a low-middle income area in São Paulo. The involvement of patients/public in the research represents a new step in the process of inclusion of all segments of our society based on patient illness and the gaps in knowledge aiming to open new horizons for continuous improvement and better acceptance of research projects.

In the first two decades of the 21st century, society has been repeatedly encouraged to be more participative in processes that might benefit populations. This global movement is directed toward building a more inclusive and egalitarian society that involves and respects minorities. Brazil has a very mixed population that faces major social inequalities. The socioeconomic disadvantage of our minority populations and of the different regions of the country is huge, particularly with regard to public health and education.

In this context, Patient and Public Involvement and Engagement (PPIE) – sometimes called Community Engagement and Involvement (CEI) – is a big challenge, but one that can be very helpful to health care professionals and stakeholders in planning better health policies to meet the main needs of the community (1,2).

PPIE in scientific research refers to an active partnership involving people from the general population and patients with community leaders or representative entities and stakeholders in a comprehensive way. Together, researchers and the community co-produce clinical research that follows scientific principles and respects community wishes (1). At first sight, researchers are seen as the main stakeholders capable of understanding the full spectrum of the disease process, the burden of symptoms, the stigma of disease, the side effects of treatment, or the barriers to accessing the health care system. However, they do not necessarily understand what is important to the community and often do not have information about illness or how participation in research is viewed from the patient’s perspective (2).

Involving patients and the public in research teams will therefore provide important information that would not be considered if only researchers were part of the team. Ideally, patients and the public need to be involved as members of the research team at all stages, from developing the research hypothesis/question to helping tailor the research by prioritizing studies about certain diseases that impact society, decreasing the individual burden of the disease. Involving patients/public in developing the research question, designing and managing the study, assisting with data collection and analysis, and disseminating the main findings from their own perspectives are just some of the important contributions envisioned (1,2).

The PPIE concept involves three pillars: public involvement, public engagement, and participation. *Public involvement* occurs when members of the general population are actively involved in developing the research question, and designing and conducting the research (2). This participation may be as co-applicants, as members of the Advisory Committee of the study, or by participating in the development of materials and questionnaires to be used in the research, such as interviewing patients to understand specific aspects of the illness that may not be as obvious to researchers. Patients/public insights into the study design also facilitate or make participation more appropriate, which helps recruitment (patients can also help with this) and retention, and ensures that outcomes are relevant to the population (2). Moreover, their participation determines how to better analyze the data considering the impact the research findings might have on the community. In addition, public involvement can help researchers understand aspects that were not previously considered, such as how disease symptoms affect patients’ daily routine, the dissemination of study results in the community, and the possible impact of these results on their lives (2).

*Public engagement* informs the public about new studies, why they are important, the impact of study results, and their possible implications in the community and in society, and deals with the dissemination of knowledge to the general population. *Participation* is being a study volunteer.

PPIE brings new perspectives to the research team to understand the disease and its burden on society, new possibilities to understand results, and empower the community and patients on all aspects of the disease. In a country as contrasting as Brazil, PPIE provides a unique opportunity to give patients/public a voice in setting research priorities and to highlight new important points to be discussed with the research team about the burden of the disease and its implications on the lives of patients and their families (2).

Our experience with PPIE, to the best of our knowledge the first initiative in Brazil, is a partnership with the University of Birmingham, the University of Liverpool, and the NIHR Global Health Group on Atrial Fibrillation (AF) Management that focuses on the AF care pathway and explores the important aspects of diagnosis and treatment in primary care in a low-to-middle-income area in the western region of the city of São Paulo (3).

In fact, we are still learning. In developing the project, we interviewed patients and community members (3). An interesting piece of information expressed by some local community members who participated in the research was about the importance of teaching the general population how to recognize AF by self-monitoring the pulse. After a physical exam showing symptoms, physicians and other health care professionals could advise individuals to self-check their pulse periodically. That way, a person can recognize that the pulse is irregular and seek medical care. The researchers noted that participants wanted to know more technical aspects of the disease. Patients raised important aspects about the burden of taking coumadin-based anticoagulants (i.e., the vitamin K antagonists, VKA) since they felt they needed to avoid green, vitamin K-rich vegetables, which would negatively impact their quality of life. They demonstrated interest in learning more about other oral anticoagulants that have fewer side effects than VKA, such as non-VKA oral anticoagulants (NOACs), even though they knew they were not offered free of charge by the Unified Health System (SUS).

How can we incorporate PPIE in research? We should try to be open-minded about these changes and ask people how they want to be involved in the research, i.e., helping the researcher reach out to new perspectives that were not previously considered in the original project, building new relationships, listening to community members, and asking them if they want to participate as co-applicants. The researcher may find people interested in participating in the study in workshops and meetings with patients and the community. Community co-applicants with equal opinion weight and rights as researchers ensure minority representation and diversity in society, which is the main goal of PPIE. Remember that it can be daunting for a member of the public/patient to voice their opinion to a group of academics and clinicians. The public/patient members need to feel relaxed, encouraged to participate, and know that their contribution will be taken seriously. Public co-applicants may bring new perspectives and experiences to the development of the study.

According to this new perspective, information from research projects must not only reach the scientific community, but also be disseminated within the community and health care systems to ensure a positive, timely impact. Involving patients/public in research represents a new step in the process of including all segments of our society, based on the patient’s illness and knowledge gaps, aiming to open new horizons for continuous improvement and better acceptance of research projects in the country.

To learn more about PPIE, see reference 2 for the PPIE Pocket Guide.
